# Variation in the Oxidative State of Collared Flycatcher (*Ficedula albicollis*) Nestlings and Its Association With Their Plumage Coloration

**DOI:** 10.1002/ece3.73835

**Published:** 2026-06-09

**Authors:** Gabriella Kőmüves, Miklós Laczi, Fanni Sarkadi, Gyula Szabó, János Török, Gergely Hegyi

**Affiliations:** ^1^ Behavioural Ecology Group, Department of Systematic Zoology and Ecology ELTE Eötvös Loránd University Budapest Hungary; ^2^ Doctoral School of Biology ELTE Eötvös Loránd University Budapest Hungary; ^3^ HUN‐REN–ELTE–MTM Integrative Ecology Research Group Budapest Hungary; ^4^ Lendület Ecosystem Services Research Group, Institute of Ecology and Botany HUN‐REN Centre for Ecological Research Vácrátót Hungary

**Keywords:** antioxidant capacity, brood size, *Ficedula albicollis*, free radicals, hatching date, visual signal

## Abstract

Plumage coloration is widely recognized as an important component in intraspecific communication across different life stages. Although coloration received a lot of attention within the field of visual communication, we have little information on whether the oxidative state influences plumage coloration during early life. Our aim was to examine the variation of oxidative parameters and their associations with coloration of two different nestling plumage traits in collared flycatchers ( *Ficedula albicollis* ). We conducted spectrophotometric measurements on the nestlings' melanin‐based wing coverts and porphyrin‐based wing covert stripe and evaluated oxidative state by measuring the amount of reactive oxygen metabolites (ROM) and the plasma antioxidant capacity (OXY). At the nest level, oxidative state parameters differed in repeatability: ROM showed low repeatability, whereas OXY was not repeatable. This pattern suggests that variation in diet and other unknown factors in the study population may influence these measures, and that the two parameters may be affected to different extents by these underlying factors. We revealed that the oxidative state deteriorated with the progression of the breeding season. Individuals that hatched in late nests had a greater amount of ROM. Furthermore, oxidative state deteriorated with brood size, as ROM levels, but only in female nestlings, increased with the number of nestlings in the brood. Considering reflectance measures, we found that when low OXY was associated with high ROM, wing covert UV chroma increased. This may suggest that individuals who experienced worse oxidative state exhibited a shift toward higher eumelanin proportion in their melanin‐based wing coloration. These findings underline the importance of oxidative balance for plumage color traits with diverse proximate backgrounds, and may encourage further investigations in the field of nestling visual traits.

## Graphical abstract

Plumage coloration is widely recognized as an important component in intraspecific communication. Our aim was to examine the variation of oxidative parameters and their associations with the coloration of two different nestling plumage traits in collared flycatchers ( *Ficedula albicollis* ). Results suggest that individuals who experience a worse oxidative state expressed less developed coloration.

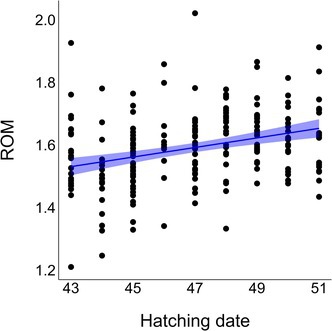

## 1 Introduction

In birds, plumage coloration can function as a visual signal that often reflects different individual attributes, providing information to conspecifics and helping to guide decision‐making in social interactions (Roulin et al. 2001; Kingma et al. 2008; de Zwaan et al. 2019). A large body of evidence suggests that variation in plumage coloration can be linked to distinct aspects of individual quality, such as condition (Guindre‐Parker and Love 2014), nutritional state and diet quality (McGraw et al. 2002; McGlothlin et al. 2007), immune state (Pardal et al. 2018), and oxidative state (Marton et al. 2022). Honesty in visual signaling can be maintained by the costs associated with the production and maintenance of these traits (Zahavi 1977; Számadó 2011). For instance, visual signals may associate with energy and resource allocation costs (Hõrak et al. 2010; Pardal et al. 2018).

Plumage coloration can arise by two major mechanisms: it can be based on pigment deposition (pigment‐based coloration) or on the structural elements of feathers (structural coloration). But these two, in most cases, form the coloration together (Shawkey and Hill 2005; Laczi et al. 2019) as pigments are embedded in structural elements and light interacts with both (Shawkey and D'Alba 2017). Pigments, such as melanins and porphyrins, contribute to coloration mainly by absorbing incident light. Melanins are among the most common integumental pigments, and their two main forms are eu‐ and pheomelanin, which have different absorption properties. Different ratios of eu‐ and pheomelanin together with feather microstructure create a range of brown, chestnut, and rusty colors (Galván et al. 2011; Shawkey and D'Alba 2017). Melanins are synthesized de novo, and the expression of these pigment‐based coloration traits can signal individual quality, such as condition (Griffith et al. 2006; McGraw 2008) or oxidative state (Galván and Alonso‐Alvarez 2008), which can facilitate intraspecific interactions in social situations, for example during mate choice (Roulin et al. 2001; de Zwaan et al. 2019). In contrast to melanins, porphyrin‐based coloration, which creates yellow, reddish‐brown, rust‐brown, purple, pink and ochre integumental coloration, received less attention in studies which focus on visual signals (Galván et al. 2018; Laczi et al. 2025, 2026). Compared to pigment‐based colors, which are based on light absorption, the formation of structural colors are generated through light scattering and interference. In this case, incident light can create broad‐spectrum, reflectance through disordered structures (e.g., in unpigmented feathers), noniridescent, constant shades, such as UV‐blue through quasi‐ordered structures, or iridescence (angle‐dependent reflectance) through laminar and crystal‐like arrays of keratin and melanosomes (Shawkey and D'Alba 2017).

Importantly, both pigment‐based and structural coloration are dynamic traits that can change over time due to various biotic and abiotic factors such as ectoparasites (Kose and Møller 1999), diet quality (McGraw 2007; Galván et al. 2018), dirt and abrasion (Surmacki and Nowakowski 2007; Surmacki et al. 2011), brood size (Hegyi et al. 2023), and UV irradiation (Surmacki 2008). In parallel, plumage coloration has been reported to correlate with individual oxidative physiology (Henschen et al. 2016; Marton et al. 2022; but see Isaksson and Andersson 2008), although studies examining this association in early life stages have yielded inconsistent results (Galván et al. 2010, 2018; Larcombe et al. 2010; Koivula et al. 2011; Arai et al. 2017). Most research has focused on carotenoid‐based coloration, for which the links with oxidative physiology are relatively well understood (e.g., Larcombe et al. 2010; Koivula et al. 2011). In contrast, substantially less is known about other coloration types, for example, melanin‐ and porphyrin‐based traits. In melanin‐based coloration, mechanisms underlying associations with oxidative state may arise through multiple, distinct pathways. One potential mechanism is genetic pleiotropy, in which shared genes can influence both pigmentation and oxidative balance, which requires a substantial genetic contribution to these traits (Ducrest et al. 2008). In parallel, coloration may be indirectly linked to oxidative state via a mechanistic pathway involving glutathione (GSH). GSH is a key antioxidant involved in both melanin synthesis and antioxidant defense (Galván et al. 2012; Galván and Solano 2016) which can be influenced by external factors, such as diet (Wan et al. 2019). This proposed mechanistic link between coloration and individual condition involves oxidative balance. When there is an imbalance between reactive oxygen species and the antioxidant defense system, in favor of the former (i.e., oxidative stress occurs), it could affect plumage coloration traits (reviewed in Metcalfe and Alonso‐Alvarez 2010) because only high‐quality individuals with better oxidative state may be able to cope with elevated free radical levels (via antioxidant capacity) and develop or maintain costly visual signals at the same time (Galván and Alonso‐Alvarez 2008; Garratt and Brooks 2012). Despite these proposed mechanisms, studies addressing how melanin‐based coloration links to oxidative state (particularly in early life stages) remain scarce (Galván et al. 2010; Roulin et al. 2011; Arai et al. 2017; Galván 2017; Rodríguez‐Martínez et al. 2019; Emaresi et al. 2022).

The association between porphyrin‐based coloration and oxidative physiology remains a particularly poorly understood topic. Porphyrin production is linked to heme synthesis (Goldberg et al. 1956), during which porphyrins act as intermediates and serve as prosthetic groups in a range of different proteins. Thus, this is a fundamentally different biosynthetic pathway compared to melanin production. However, certain types of porphyrins may be associated with oxidative processes involving free radicals. For example, Galván et al. (2018) found that feather coproporphyrin III concentration was positively associated with body mass and breeding site quality in Eurasian eagle owl ( *Bubo bubo* ) fledglings. As this porphyrin form can be produced under oxidative stress, they suggest that coproporphyrin III may signal breeding site‐mediated capacity to cope with oxidative stress to conspecifics. A potential relationship may therefore exist between porphyrin‐based coloration and oxidative state through free‐radical‐mediated processes, whereby the expression of coloration may reflect the individual's ability to cope with oxidative stress.

Various aspects of plumage coloration are known to be shaped by sexual selection in adult birds (Andersson 1994), and accordingly, the vast majority of studies have focused on adult individuals. However, plumage coloration also varies among individuals in early life stages (Hõrak et al. 2000; García‐Campa et al. 2023). Generally, nestlings show cryptic coloration, which can provide fitness advantages in the nest (reviewed by Kilner 2006), but individual variation in coloration and its function has gained less attention. Nevertheless, variation in nestling coloration can be driven by different selection processes, such as parental favoritism (Lessells 2002; Romano et al. 2016; Costanzo et al. 2017). Previous studies suggested that plumage coloration developed in the nestling stage can signal the individual's sex (Siefferman et al. 2008; Kapun et al. 2011), condition (Johnsen et al. 2003; Jacot and Kempenaers 2007), and resistance to parasites (Surmacki et al. 2015). In addition, experiments revealed that different aspects of the rearing environment, such as brood size, the amount of parental care, and resource availability can have a substantial effect on plumage coloration in nestlings (Siefferman and Hill 2007; Jacot and Kempenaers 2007; Musgrove and Wiebe 2016), and on oxidative state also (Costantini and Dell'Omo 2006a; Losdat et al. 2010; Bourgeon et al. 2011; López‐Arrabé et al. 2016). For example, in collared flycatcher nestlings, previous results revealed sex differences in both melanin‐ and porphyrin‐pigmented plumage parts (Laczi et al. 2025). Both pigment types were also found to be associated with environmental conditions, although they differ in their production pathways and sensitivity to environmental factors (Laczi et al. 2026). These results suggest that plumage coloration may play a role in communication during this early life stage.

Studying the function of plumage color variation in the nestling stage can be highly relevant for understanding parent–offspring communication and social interactions among siblings. As an adaptive visual signal, nestling coloration may mediate competition for parental care among nestlings, simultaneously allowing parents to adjust their allocation strategies among offspring, as coloration may reflect individual differences, for instance in future survival prospects. By responding to such cues, parents may direct a greater degree of parental investment toward more valuable offspring (i.e., more intensely colored ones) to increase their own fitness (Lyon et al. 1994; Lessells 2002; Krebs and Putland 2004; Romano et al. 2016; Costanzo et al. 2017).

Here, our aim was to investigate the information content of nestling wing coverts coloration with respect to oxidative state parameters (i.e., antioxidant capacity and oxidative damage) in a natural population of collared flycatchers. The relationship between reflectance and oxidative state has not previously been investigated during the early life stages of this species, thus it remains unknown whether oxidative state may contribute to information conveyed by plumage traits during social interactions with parents or siblings. In addition, in the wing coverts, most of the feathers developed in the nestling period remain until the first breeding attempt (Demongin 2016), although the yellow porphyrin‐based pigmentation is lost in yearlings, with the feathers appearing white due to photodegradation of the originally deposited porphyrin compounds, suggesting a role in communication not only in early life stages, but also during the first breeding. In our study, we analyzed coloration traits of two plumage parts, namely nestlings' wing coverts, which are pigmented by melanin, and the wing covert stripe, which is pigmented by porphyrin (Laczi et al. 2026). We also aimed to investigate the effects of rearing environment and sex on nestlings' oxidative state, and whether the oxidative state is an individual‐specific trait or characteristic to the entire brood.

Factors, such as hatching date (López‐Arrabé et al. 2016), brood size and sibling competition (Bourgeon et al. 2011), can influence the quantity and quality of available resources, and consequently individuals' oxidative state (Giordano et al. 2015). Dietary intake provides components with antioxidant properties as well as nutrients that can be used to build various antioxidant molecules (Catoni et al. 2008; Lee et al. 2013). We predicted that hatching date would influence oxidative state, with later‐hatched nestlings expected to have poorer oxidative state. This is because food availability typically changes throughout the season and declines after the peak period (Buse et al. 1999) and this reduced availability may constrain nestlings' development (Fargallo et al. 2007; Garrido‐Bautista et al. 2021) and oxidative state (López‐Arrabé et al. 2016). Similarly, we assume that brood size would affect oxidative state. Nestlings from larger broods are expected to have worse oxidative state as increased sibling competition can also reduce available resources (Bourgeon et al. 2011). In our population, we previously found that broods that miss the food peak are disadvantaged in terms of growth (Hegyi et al. 2013), thus it is plausible that this may have a negative effect on oxidative state as well, as individuals may be unable to acquire sufficient quantity and quality of food to support an adequate antioxidant defense system.

Nestling wing coloration has previously been shown to be related to the rearing environment, with less favorable conditions (bigger brood size, mismatch with food peak) associated with less developed wing coloration (i.e., brown plumage part was less brown and brighter, while yellow part was less yellow and less bright, Laczi et al. 2026). We also expected an association between oxidative state and coloration, for both melanin and porphyrin‐based traits. For melanin‐based coloration, a potential link with oxidative state can be mediated by GSH availability or pleiotropic effects (see above); therefore, we predicted that nestlings in better oxidative state would exhibit more pronounced wing coloration with lower UV chroma and brightness. In contrast, the relationship between porphyrin‐based coloration and oxidative state is less understood; however, the production of certain porphyrin forms are involved in oxidative processes related to free radicals (Woods and Calas 1989; Galván et al. 2018). Accordingly, we expected porphyrin‐based coloration at this stage to be associated with the bearer's oxidative state, which is influenced by environmental conditions. We expected more pronounced yellow coloration, characterized by higher saturation and brightness, in individuals with a better oxidative state.

## 2 Materials and Methods

### 2.1 Study Site and Species

This study was carried out in 2023 in a long‐term study plot in the Pilis‐Visegrádi Mountains, near Pilisszentlászló (Duna‐Ipoly National Park, Hungary, 47.725 N, 19.006 E). The nest box plots are situated in a deciduous oak‐hornbeam woodland, and they cover approximately 38 ha. The study area contained 441 B‐type nest boxes arranged in a grid system at the time of the study, which are mainly used by collared flycatchers, and to a lesser extent by great tits ( *Parus major* ) and blue tits ( *Cyanistes caeruleus* ). The average distance between nest boxes is 30.7 m. The collared flycatcher is an insectivorous, hole‐nesting, long‐distance migratory passerine. Females lay and incubate 3–8 (mostly 5–7) eggs. Both parents participate in the caretaking of nestlings, which are fed a diverse diet in which folivorous caterpillars represent a major component (Chaplyhina et al. 2022). Young birds leave the nest after the age of 13–14 days. Nestlings' abdominal plumage is characterized by whitish coloration with melanin pigmented brown patterns. Their dorsal part is predominately brown with distinct yellow spots on the head and along the back. In addition, nestlings are ornamented with yellow tips on their brown greater coverts, which form a continuous stripe in their wings. It has been shown that this yellow stripe coloration is produced by porphyrin pigments in the feathers (Laczi et al. 2026). Additionally, sex‐related differences were found in the brightness and UV chroma of the primary coverts, as well as in wing covert stripe coloration. The first molt occurs in the breeding site, prior to the migration to Africa and involves the replacement of body feathers only. This is followed by a prebreeding molt during the winter in Africa; however, the primaries and some of the greater coverts are not included in this molt either, being retained until the first breeding attempt (Demongin 2016). In yearlings, previously yellow plumage parts are already white, most likely due to photobleaching, as porphyrins are highly photodegradable (Bezdetnaya et al. 1996).

### 2.2 Field Methods

We checked all nest boxes every fifth day to record breeding parameters, including hatching date (date of the first nestling hatching in the nest, relative to April 1) and the number of nestlings that hatched in the nest (hereafter, brood size). Nestlings were taken from the nest boxes on two different occasions, with handling time minimized as much as possible. Prior to all measurements, nestlings were kept in a containment bag. At the age of 8 days, corresponding to the peak period of feather growth (Järvinen and Ylimaunu 1984), we ringed nestlings and collected blood samples from the brachial vein. It is this peak growth time when oxidative processes are most informative of feather development, as rapid feather growth can be energetically demanding, thereby elevating metabolic rate and influencing individuals' oxidative state (López‐Arrabé et al. 2016). Thus, the oxidative state measured at this time well reflects the physiological constraints affecting feather formation. Blood was collected into two Na‐heparinised microcapillary‐hematocrit tubes (for molecular sexing and oxidative state determination) for each individual. For molecular sexing, blood was stored in absolute ethanol at −20°C until DNA extraction (see below). The tubes for oxidative state determination were stored at 0°C–5°C and centrifuged within 2 h (10,000 rpm for 10 min) to separate plasma from blood cells, after which plasma samples were stored at −20°C until analysis. At the nestling age of 13 days, we measured body mass using a Pesola spring balance (with a precision of 0.1 g) and tarsus length with a caliper (with a precision of 0.1 mm) and also performed spectrophotometric measurements.

### 2.3 Spectrophotometric Measurements

We measured the reflectance spectra of the nestlings' wing coverts and wing covert stripes. We used a USB2000 spectrophotometer with a DH2000 light source and an R400‐7 bifurcated fiber optic probe (Ocean Optics Europe). For each bird, we took two reflectance samplings within a plumage part, primarily for ethical and logistical reasons. The probe was removed and repositioned between the two measurements. To standardize distance and keep out ambient light, the probe had a black plastic cover, and it was positioned perpendicularly to the feather surface. Reflectance was calculated relative to a white WS‐1 diffuse reflectance standard (Ocean Optics Europe) and a dark reference (no light reached the detector). Reflectance values were recorded with the OOIBase32 software (Ocean Optics Europe). Using these spectra, we quantified different color traits. For the melanin‐based wing coverts, we used brightness, which defines the average reflectance in the bird‐visible range (320–700 nm), and UV chroma (mean reflectance from 320 to 400 nm divided by brightness). Even though UV chroma is often used as a variable for structurally‐based and glossy feathers, variation in relative UV reflectance may be associated with melanin composition, feather structure and condition (Siitari and Huhta 2002; Mennill et al. 2003; McKinnon and Robertson 2008; D'Alba et al. 2014). In addition, in our study population, we previously found that variation in wing covert UV chroma can be linked to sex‐specific differences in nestlings (Laczi et al. 2025), and that the rearing environment also predicts this spectral variable (Laczi et al. 2026). Additionally, we also calculated brown chroma, defined as the average intensity between 500 and 700 nm, divided by brightness. Given the strong correlation between UV chroma and brown chroma after controlling for sex (*r* = −0.963), and the high similarity of model results between the two color variables, results for brown chroma are reported in the Table S1. For the yellow stripe, we calculated brightness and relative height ([maximum reflectance − minimum reflectance]/average intensity) (Örnborg et al. 2002; Szigeti et al. 2007), which reflects the saturation of this color (hereafter referred to as saturation). We used Microsoft Excel to manage reflectance curves and to calculate the above detailed spectral traits.

### 2.4 Blood Sample Processing

#### 2.4.1 Molecular Sex Determination

For molecular sex determination, DNA was extracted by an ammonium‐acetate method (Nicholls et al. 2000). We used primers 2550F and 2718R (Fridolfsson and Ellegren 1999) and followed the PCR protocol described in Rosivall et al. (2004). PCR products were visualized by gel electrophoresis on pre‐stained (GelGreen) 2% agarose gels to determine sex.

#### 2.4.2 Oxidative State Determination

We define two parameters connected to the oxidative state of individuals, namely the amount of reactive oxygen metabolites (hereafter, ROM) and plasma antioxidant capacity (hereafter, OXY). ROM measures hydroperoxides, the early products of reactive oxygen species‐driven peroxidation of macromolecules, such as proteins. OXY indicates the capability of the antioxidant defense system to cope with the effects of free radicals by quantifying the amount of hypochlorous acid neutralized by the system (Costantini 2010; Galván 2022). We used the d‐ROMs test and OXY‐Adsorbent test respectively (Diacron International, Grosseto, Italy) to measure these physiological traits (with modifications, based on Costantini et al. 2006; Costantini and Dell'Omo 2006b; Markó et al. 2011).

In d‐ROMs test, we diluted 5 μL plasma with 200 μL of a solution containing 0.01 M acetic acid/sodium acetate buffer (pH‐value 4.8) and N,N‐diethyl‐p‐phenylenediamine as chromogen, and incubated it at 37°C for 75 min. The amount of a colored (pink) reaction product is proportional to the amount of ROMs in the sample. Absorbance was measured with a BioTek ELX808 microplate reader at 490 nm, and the concentration was estimated based on a standard solution's absorbance curve (Costantini and Dell'Omo 2006b).

In the OXY‐Adsorbent test, contrary to the prescribed protocol, we used 2 μL plasma instead of 5 μL due to the insufficient amount of blood samples from nestlings. First, we diluted 2 μL plasma with distilled water in the ratio of 1:100. Then, 2 μL from the diluted plasma was incubated with 200 μL aliquot of HOCL‐titrated solution for 10 min at 37°C. After the incubation period, we added 2 μL chromogen (it was the same compound that we used in the d‐ROMs test). Here, the intensity of the pink color is inversely related to the plasma antioxidant capacity. Absorbance was measured with the same instrument at 540 nm.

### 2.5 Statistical Methods

For statistical analyses, we only included data from nests where at least half of the nestlings had oxidative state samples, and used only those individuals from which all oxidative parameters were available. This resulted in a final dataset of 217 individuals from 44 nests. All of the individuals having complete oxidative state data also had spectrophotometric measurements. Individuals were not included in the analyses if both oxidative parameters (194 nestlings), or either ROM (9 nestlings) or OXY (21 nestlings) could not be measured because the available blood samples were insufficient in quantity or quality to allow reliable assay.

After examining the raw distributions of ROM and OXY values, we log‐transformed these parameters to approach normal distributions. We tested the repeatability of the oxidative state parameters, adding sex as a fixed correction variable and nest ID as a random factor. Repeatability was assessed using the “rpt” function of the “rptR” package (Stoffel et al. 2017). We conducted linear mixed‐effect models with nest ID as a random factor using the “lmer” function from the “lmerTest” package that applies Satterthwaite correction for the estimation of error df's (Kuznetsova et al. 2017). In all models, we applied backward stepwise model simplification, excluding nonsignificant terms from the full model (which contained all variables and interaction terms) in decreasing order of statistical significance, with significance assessed based on *p*‐values (*p* > 0.05 were removed). In the final models (which contain only the significant variables and the nonsignificant component variables of significant interactions), we reentered nonsignificant terms to check their nonsignificance in the environment of the reduced model (characterized by lower statistical noise) and to enable the calculation of parameter estimates for these terms in future meta‐analyses. After this validation, only statistically significant variables were retained in the models. The statistical significance of interactions represented a special problem in this analysis. As we described above, the sample size of actual statistical tests was affected by missing data. Moreover, the calculation of error degrees of freedom in mixed models with the Satterthwaite method depends on differences in the number of data and ratios of within‐ and among‐brood variances, and the variance explained by brood (used as a random effect here) is generally small for nestling color variables (Laczi et al. 2025). With a weak random factor, the correction of error df's is less predictable, making the exact significance level uncertain. Therefore, when encountering marginally nonsignificant interactions (0.05 < *p* < 0.1), we calculated effect sizes (Pearson r) following McNeil et al. (1996), and in case of nonnegligible effect size, we ran follow‐up analyses for groups of the corresponding fixed factor as for a significant interaction. For comparison, we also tell the effect size for significant interactions. Standard model diagnostics (histogram plotting and simple linear models) were performed to ensure that assumptions of normality, homoscedasticity and independence were met. All analyses were performed in R 4.3.2 (R Core Team 2023). Data visualization was performed using “ggplot” from the “ggplot2” package (Wickham 2016) and base R function “persp” (R Core Team 2023).

In both the oxidative state and the plumage coloration models, sex and hatching date were included as correction variables due to their previously reported effects on color traits (Laczi et al. 2025, 2026). In the models of oxidative state, the dependent variable was log‐transformed ROM or OXY, and explanatory variables were sex, hatching date, and brood size. In addition, we included the interactions of hatching date with sex and brood size with sex. For color variables, we ran four separate models. In each model, one of the four plumage color traits (covert brightness, covert UV chroma, stripe brightness, stripe saturation) was included as a dependent variable. In all four models, explanatory variables were sex, hatching date, oxidative state parameters (OXY, mean ROM and ROM deviation), and the interactions of oxidative parameters with sex. In addition, three‐way interactions among sex, OXY, and ROM were also tested. We used a within‐subject centering approach for ROM (Van de Pol and Wright 2009) because nest explained a significant part of the variance. We included both nest‐level mean values and individual deviations from the mean (hereafter, mean ROM and ROM deviation, respectively) as explanatory variables. With regard to wing covert stripe saturation, one individual was excluded from the model because visual inspection and Cook's distance indicated that it was an influential point, namely a male nestling with extremely low saturation (results for the model including the individual are provided in Table S2).

## 3 Results

We found differences in the nest‐level repeatability of oxidative state parameters, more specifically ROM had low repeatability (intraclass correlation (*R*); *R* = 0.206, 95% CI = 0.061–0.345, *p* < 0.001), while OXY values were not repeatable (*R* = 0.03, CI = 0–0.127, *p* = 0.287).

**FIGURE 1:**
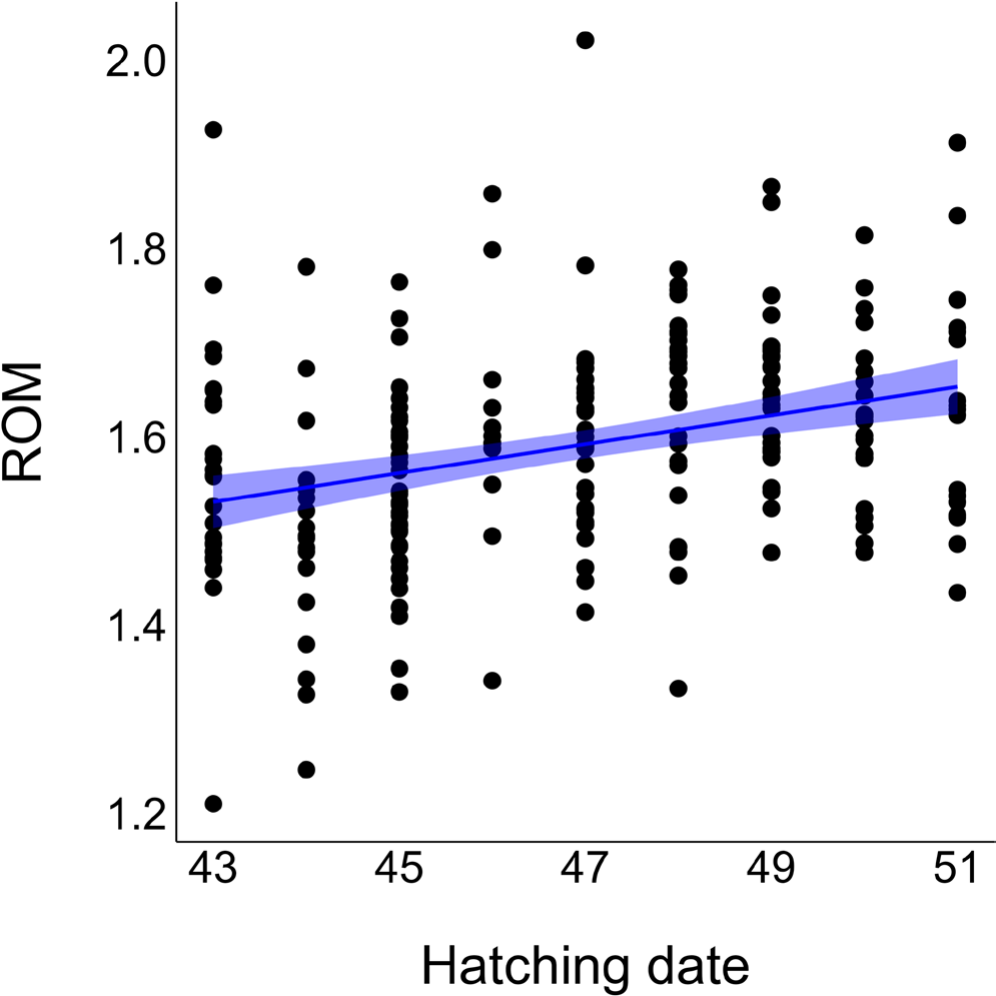
Association between ROM and hatching date. ROM defines the amount of reactive oxygen molecules. Hatching date was expressed as the number of days passed since 1 April. Log‐transformed ROM values are presented in this figure. Shading represents the 95% confidence interval around the fitted line.

**FIGURE 2:**
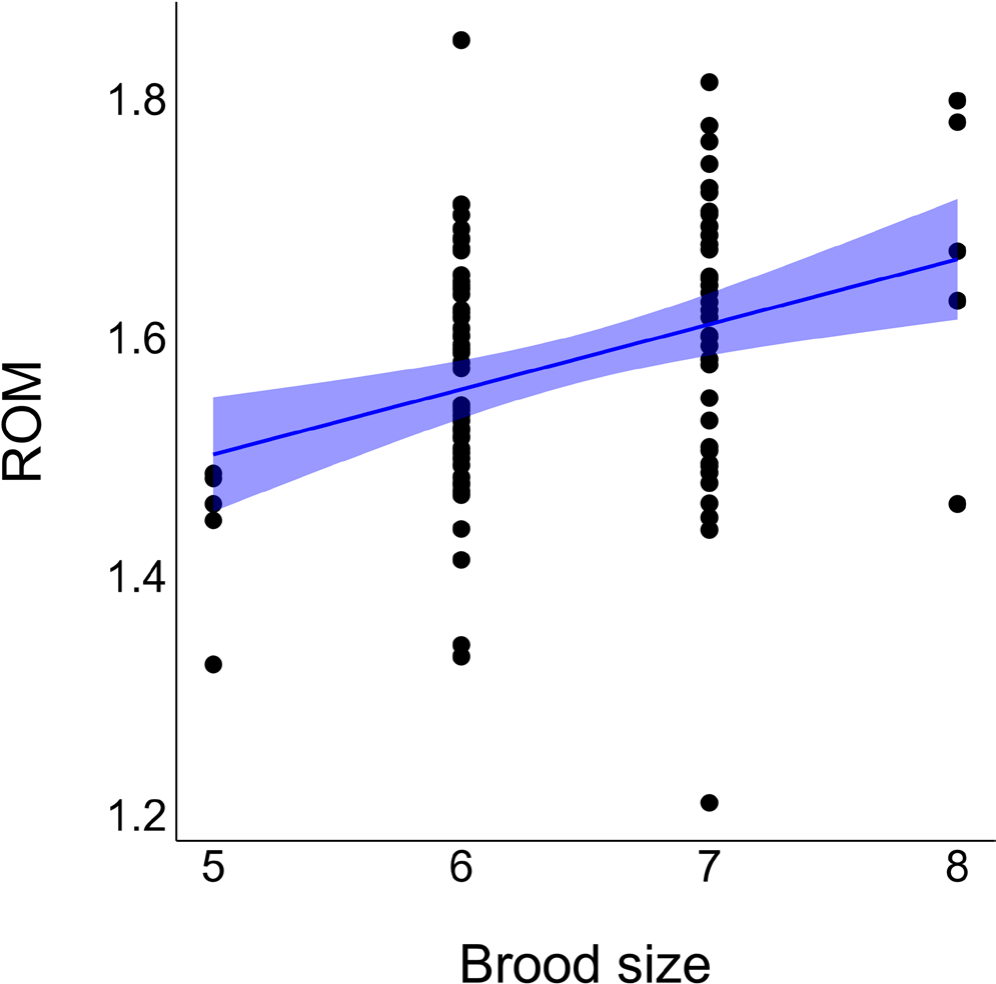
Association between ROM and brood size in females. ROM defines the amount of reactive oxygen molecules. Brood size represents the number of individuals hatched in the nests. Log‐transformed ROM values are presented in this figure. Shading represents the 95% confidence interval around the fitted line.

We found that ROM increased with hatching date, indicating that nestlings from later‐hatching broods had worse oxidative state (see Table 1 and Figure 1). In addition, brood size had a significant main effect, while results revealed a marginal interaction with sex (*p* = 0.0504) with an effect size of *r* = 0.139. We performed separate exploratory follow‐up analyses for males and females. We found that ROM increased with brood size, in female nestlings (*β* ± SE = 0.065 ± 0.016, df1 = 1, df2 = 39.73, *F* = 16.65, *p* < 0.001, Figure 2.), whereas no such association was detected in males: (*β* ± SE = 0.021 ± 0.021, df1 = 1, df2 = 42.77, *F* = 0.96, *p* = 0.332). Results showed no significant association between OXY and any of the investigated variables (see Table 1), only a marginal interaction between sex and hatching date (*p* = 0.061) with an effect size of *r* = 0.130. We conducted follow‐up analyses for males and females separately to explore the aforementioned tendency, but there were no significant effects of hatching date in either sex (females: *β* ± SE = −0.007 ± 0.006, df1 = 1, df2 = 102, *F* = 1.74, *p* = 0.19; males: *β* ± SE = 0.008 ± 0.006, df1 = 1, df2 = 111, *F* = 1.81, *p* = 0.182).

**TABLE 1:** Results of the analyses of nestlings' oxidative state.

	ROM				OXY			
	*β* (SE)	df1	df2	*F*	*β* (SE)	df1	df2	*F*
Sex	−0.011 (0.014)	1	192.05	0.63	0.029 (0.020)	1	215	2.09
Hatching date	**0.016 (0.004)**	**1**	**40.94**	18.96***	0.000 (0.004)	1	47.48	0.00
Brood size	**0.043 (0.014)**	**1**	**43.74**	9.11**	0.013 (0.016)	1	52.82	0.67
Sex × hatching date	0.000 (0.006)	1	194.92	0.00	−0.015 (0.008)	1	206.33	3.54^†^
Sex × brood size	0.043 (0.022)	1	196.11	3.88^†^	0.025 (0.031)	1	213	0.68

*Note:* ROM defines the amount of reactive oxygen molecules, whereas OXY expresses plasma antioxidant capacity. Both ROM and OXY values were log‐transformed prior to the analyses. Effects included in the final models are highlighted in bold.

^†^ *p* < 0.10, *p < 0.05, **p < 0.01, ***p < 0.001.

**FIGURE 3:**
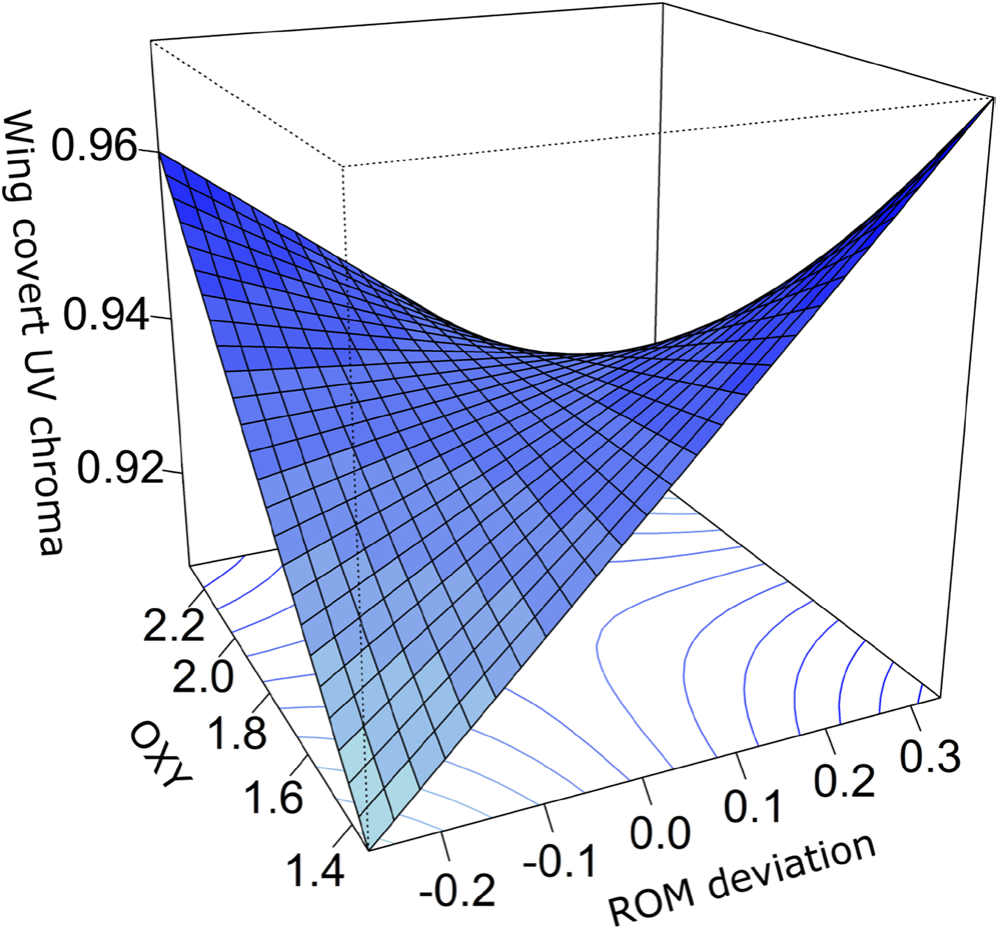
Association between wing covert UV chroma and oxidative state parameters. OXY represents plasma antioxidant capacity, while ROM deviation indicates the deviation from the nest‐level mean of reactive oxygen metabolites. Log‐transformed ROM and OXY values are presented in this figure.

Regarding plumage coloration, wing covert UV chroma was higher specifically in individuals with low OXY and high ROM deviation (significant OXY × devROM interaction with an effect size of *r* = 0.142, see Table 2, Figure 3). The interaction between sex and ROM deviation, on the other hand, had a marginal effect (*p* = 0.070 with an effect size of *r* = 0.125) on covert stripe saturation (see Table 2). We again conducted follow‐up exploratory analyses by sex and found a marginal negative association in females, while the result was far from significant in males (females: *β* ± SE = −0.337 ± 0.176, df1 = 1, df2 = 96.93, *F* = 3.68, *p* = 0.058; males: *β* ± SE = 0.120 ± 0.143, df1 = 1, df2 = 84.51, *F* = 0.71, *p* = 0.403). Oxidative state measures, however, showed no relationship with either covert brightness or stripe brightness, and the interaction terms were likewise nonsignificant.

**TABLE 2:** Results of the analyses of nestlings' plumage color traits.

	Wing covert brightness				Wing covert UV chroma				Wing stripe brightness				Wing stripe saturation			
	*β* ± SE	df1	df2	*F*	*β* ± SE	df1	df2	*F*	*β* ± SE	df1	df2	*F*	*β* ± SE	df1	df2	*F*
Sex	**0.573 ± 0.203**	**1**	**199.02**	7.99**	**−0.019** ± **0.004**	**1**	**197.27**	19.53***	**−1.629** ± **0.509**	**1**	**188.64**	10.24**	**0.041** ± **0.020**	**1**	**188.61**	4.10**
Hatching date	0.009 ± 0.045	1	39.63	0.04	−0.001 ± 0.001	1	45.25	1.16	**0.450** ± **0.141**	**1**	**40.05**	10.19**	**0.014** ± **0.006**	**1**	**41.47**	6.54**
OXY	0.378 ± 0.697	1	212.13	0.29	**0.008** ± **0.015**	**1**	**208.25**	**0.27**	−0.456 ± 1.773	1	200.63	0.66	−0.098 ± 0.070	1	199.10	1.94
Mean ROM	−1.223 ± 1.470	1	39.78	0.69	−0.021 ± 0.033	1	44.52	0.42	−8.411 ± 5.229	1	41.53	2.59	−0.189 ± 0.211	1	41.53	0.80
ROM deviation	−0.063 ± 1.103	1	169.03	0.00	**0.507** ± **0.245**	**1**	**202.82**	4.27*	−1.748 ± 2.711	1	168.63	0.42	−0.044 ± 0.107	1	169.32	0.17
Sex × OXY	−1.302 ± 1.394	1	201.03	0.87	−0.027 ± 0.030	1	199.03	0.81	−0.425 ± 3.535	1	188.57	0.01	−0.152 ± 0.139	1	186.67	1.21
Sex × mean ROM	0.852 ± 2.673	1	193.05	0.10	−0.000 ± 0.055	1	190.52	0.00	2.325 ± 6.662	1	185.69	0.12	0.104 ± 0.264	1	184.27	0.16
sex × ROM deviation	−1.060 ± 2.312	1	211.18	0.21	−0.020 ± 0.050	1	210.97	0.16	−1.076 ± 5.964	1	208.91	0.03	−0.427 ± 0.235	1	208.52	3.31^†^
OXY × mean ROM	3.761 ± 9.310	1	208.23	0.16	−0.125 ± 0.195	1	202.59	0.41	19.013 ± 23.504	1	197.19	0.65	−0.724 ± 0.924	1	192.92	0.61
OXY × ROM deviation	−1.710 ± 6.462	1	205.86	0.07	**−0.273** ± **0.133**	**1**	**203.07**	4.20*	−0.500 ± 16.374	1	190.65	0.00	0.014 ± 0.642	1	188.23	0.00
Sex × OXY × mean ROM	4.162 ± 9.342	2	204.09	0.30	−0.114 ± 0.196	2	200.15	0.44	19.081 ± 23.615	2	193.59	0.33	−0.675 ± 0.929	2	189.60	0.51
Sex × OXY × ROM deviation	−1.496 ± 6.491	2	208.35	0.14	−0.008 ± 0.027	1	210.96	0.10	−0.184 ± 16.485	2	198.94	0.02	0.154 ± 0.642	2	197.42	2.16

*Note:* OXY represent plasma antioxidant capacity. Mean ROM was defined as the nest‐level mean amount of reactive oxygen metabolites, whereas ROM deviation indicates the within‐nest deviation from it. Both ROM and OXY values were log‐transformed prior to the analyses. Effects included in the final models are highlighted in bold.

^†^ *p* < 0.10, *p < 0.05, **p < 0.01, ***p < 0.001.

## 4 Discussion

In this study, we investigated the relationship between oxidative state parameters and plumage coloration in nestling collared flycatchers. We also examined whether certain aspects of the rearing environment were associated with oxidative state during early life stages. Our results indicate that oxidative state parameters are associated with wing covert UV chroma, while various components of the rearing environment (such as hatching date in both sexes and brood size in females) were linked to oxidative state in early life stages. Our findings suggest that some aspects of early‐life environment affect oxidative state and that plumage coloration may convey information about individual oxidative state.

We found that oxidative‐state‐related parameters differed in terms of nest‐level repeatability. Namely, ROM showed low repeatability, while OXY was not significantly repeatable. These patterns suggest that siblings were weakly similar to each other with respect to ROM, but in contrast to this, there were no signs of such similarity in the case of OXY. There are several possible explanations for the low or nonsignificant repeatability observed for the oxidative parameters. First, oxidative state can be strongly influenced by diet; thus, if nestlings within the same brood receive qualitatively or quantitatively different food intake from parents, this dietary heterogeneity could reduce nest‐level repeatability. For example, dietary vitamin intake has been shown to affect the antioxidant defense system of great tit ( *Parus major* ) nestlings (Marri and Richner 2014), while in nestling pigeons ( *Columba livia* ) food quality influenced both serum hydroperoxide levels and antioxidant responses (Costantini 2010). Second, a common environment effect of brood size‐related competition may tend to homogenize nestlings within broods in terms of stress and therefore potentially ROM. Indeed, physiological stress was found to increase with manipulated brood size in the sister species pied flycatcher (Ilmonen et al. 2003). A partial support for this effect is provided by the association of brood size and ROM in female nestlings in our study. Competition‐related stress may specifically increase repeatability for ROM (more stress‐dependent) but not for OXY (more nutritionally dependent), thereby potentially explaining our results. Previously, Costantini and Dell'Omo (2006a), using a cross‐fostering experiment in Eurasian kestrel ( *Falco tinnunculus* ) nestlings, found that different factors influenced the variance of the two oxidative parameters. Their results suggest that variation in ROM levels appears to show stronger genetic effects, whereas variation in OXY values may predominantly reflect environmental factors, such as dietary differences. In contrast, Losdat et al. (2014) found that oxidative state parameters had a low additive genetic basis and were highly influenced by common environmental effects. Thus, taken previous findings together, our results may suggest that ROM and OXY may be differently shaped by genetic and varying level environmental factors. While ROM values may be more strongly affected by shared genetic or maternal effects, OXY may reflect individual‐specific effects. Importantly, similar genetic influences as in ROM may also be present for OXY, but these could be masked at the nest level due to stronger environmental effects, resulting in low repeatability. The mechanistic background of these differences therefore needs further studies.

Considering the determinants of oxidative state, no association was revealed between antioxidant defense strength (OXY) and any of the investigated explanatory parameters. On the other hand, individuals that hatched in late nests had a greater amount of ROM. The increase of ROM implies that oxidative state significantly deteriorated with hatching date. In addition, brood size also had a significant association with nestlings' oxidative state in females, as ROM levels of this sex increased with the number of individuals hatched in the nest. In the postnatal stage, nestlings have an immature antioxidant defense system; hence, development and fast growth can be highly demanding to them (López‐Arrabé et al. 2016). In this period, the antioxidant defense system can be improved by adequate nutritional care (Blount et al. 2003). However, if nutritional requirements are not fulfilled in terms of quality or quantity (due to the deteriorating environmental factors by the end of the breeding season or because of high levels of sibling competition such as in a large brood), an imbalance may occur between the amount of free radicals and the antioxidant defense system, which can ultimately lead to oxidative stress. For example, in the closely related pied flycatcher ( *Ficedula hypoleuca* ), associations have been found between antioxidant defenses of nestlings and hatching date (López‐Arrabé et al. 2016), while in European starlings ( *Sturnus vulgaris* ), brood size negatively affected antioxidant capacity in one of two study years (Bourgeon et al. 2011). In our study population, previous results suggested that asynchrony with the caterpillar peak had a negative effect on nestlings' development (Hegyi et al. 2013), as well as on their plumage coloration (Laczi et al. 2026). Thus, it is plausible that conditions experienced during early life can also affect nestlings' oxidative state in this species. On the other hand, sex‐specific differences also exist in how nestlings respond to environmental conditions. Although previous studies have primarily documented sex‐related differences in antioxidant capacity, for instance in great tit (De Coster et al. 2012) or pied flycatcher (López‐Arrabé et al. 2016) nestlings, it is possible that such differences may also appear in other components of oxidative state. In this species, females may therefore be more susceptible to the negative effects of increased brood size, potentially leading to elevated levels of ROM.

We found an association between oxidative state and plumage coloration in collared flycatcher nestlings, indicating that correlation between certain plumage traits and oxidative state is not only present in adults (Markó et al. 2011), but a similar association could already be found in nestlings. UV chroma of the brown wing coverts increased with ROM, particularly in nestlings with low OXY. Therefore, nestlings experiencing oxidative stress exhibited wing coverts with higher UV chroma. Nestlings' brown coloration is likely the result of two types of melanin pigments, namely eu‐ and pheomelanin, which are present together in most of the melanin‐colored plumage patches (McGraw et al. 2005). Melanins exhibit broad wavelength range absorbance (Tran et al. 2006; Galván and Solano 2016), but pheomelanin may be more prone to absorb in the UV‐A range (Tran et al. 2006; Riesz 2007). Accordingly, UV chroma may provide information about the ratio of eumelanin to pheomelanin, with higher values potentially indicating a greater proportion of eumelanin. Indirect support for our predictions may be provided by the study of Calhim et al. (2014), who investigated plumage coloration in adult pied flycatchers. In that study, males and females differed not only in melanin composition but also in UV chroma values, with higher UV chroma being associated with a higher relative contribution of eumelanin. Alongside the distinct absorption properties of different melanin pigments (Galván 2022), structural properties of feathers, independent of pigment composition, can also influence UV chroma values (Laczi et al. 2019). Consequently, given that our knowledge on how UV chroma is precisely related to pigment composition is currently limited in our study population, further studies are required to clarify the mechanisms underlying this association.

The pattern we detected between plumage coloration and oxidative state can arise due to multiple, mutually nonexclusive reasons. The link between melanogenesis and oxidative state can itself be mediated by multiple physiological processes. First, a possible mechanism is genetic pleiotropy, as some genes may influence both oxidative balance and plumage pigmentation (Wu et al. 2004; Galván et al. 2017; San‐Jose and Roulin 2018). For example, the *Slc7a11* gene encodes a protein that mediates cysteine transport into melanocytes, which is essential for pheomelanin synthesis. As cysteine is a key precursor of GSH, this allocation may reduce GSH availability for the antioxidant defense system (Galván et al. 2017; San‐Jose and Roulin 2018). Additionally, a biochemical connection is also possible between melanin synthesis and antioxidant defense through GSH. For eumelanogenesis to occur, high tyrosinase activity and low cysteine levels are required, whereas high cysteine levels and low tyrosinase activity lead to the production of pheomelanin. GSH is one of the most important intracellular antioxidants and it also regulates melanogenesis (Galván et al. 2010). As GSH can indirectly inhibit tyrosinase enzyme activity and raise cysteine level, a high level of GSH can shift the process toward pheomelanin production (Galván et al. 2010, 2012). In our case, higher oxidative stress may be associated with higher eumelanin content, which could reflect a trade‐off between the antioxidant‐defense system and plumage coloration. When antioxidant capacity is low, higher ROM is associated with increased UV chroma, which indicates an increase in the proportion of eumelanin. It is plausible that limitations in the quality or quantity of available resources within broods may reduce the availability of GSH. Given the limited availability of GSH, a negative shift in oxidative state may direct the available GSH to reinforce antioxidant defenses, while pigment production shifts toward the less GSH‐demanding pathway, that is, eumelanogenesis (Galván and Alonso‐Alvarez 2008). We have previously shown that nestlings reared under relatively unfavorable conditions developed brown wing coverts with higher UV chroma (Laczi et al. 2026). Thus, melanin‐based wing coverts may reflect individual quality and potentially also the ability to cope with oxidative stress. Only high‐quality individuals may be able to invest in increased mobilization of other antioxidants and develop plumage with higher pheomelanin content simultaneously, while GSH levels are low. In contrast, individuals with a more limited capacity to cope with oxidative stress may rely on the production of less costly melanin‐based coloration, that is, eumelanin‐based plumage, thereby conserving GSH for its role in antioxidant defense. However, further studies are necessary to investigate the exact mechanism.

Concerning the wing covert stripe, in female nestlings we detected a weak negative association between saturation and ROM deviation, which means that individuals with poorer oxidative state relative to the brood mean tended to develop paler yellow color. These findings may have resulted from reduced pigment content, leading to lower saturation. In collared flycatcher nestlings, the temporary yellowish color of the wing stripe is created by porphyrin pigment deposition, which has now been demonstrated based on the combination of the reflectance curve shape and the presence of UV‐induced fluorescence in this plumage area (Laczi et al. 2026). Porphyrins are common pigments produced during the synthesis of heme (Goldberg et al. 1956) that can contribute to plumage coloration in birds. Despite the contribution and potential function of these pigments (Galván et al. 2016), the information content of such color traits remains poorly investigated. However, a few studies have previously suggested that feather porphyrin content may function as a visual signal in young birds. In Eurasian eagle owl fledglings, amount of one of the porphyrin forms detected from body feathers was positively associated with body mass and breeding site quality (Galván et al. 2018). Another example is provided by Camacho et al. (2019), where variation in porphyrin‐based coloration was associated with body condition in juvenile red‐necked nightjars ( *Caprimulgus ruficollis* ). However, the link between porphyrin content and oxidative state remains unclear. While possible pathways involving heme synthesis have been proposed, current evidence is primarily limited to cellular‐level mechanisms (e.g., nonenzymatic oxidation by free radicals, Woods and Calas 1989; Galván et al. 2018) rather than direct links to plumage. In addition, it is important to emphasize that this pigment type is highly photodegradable (Moan 1988; Ericson et al. 2003). Consequently, the plumage area may turn into white (when porphyrin is the only pigment present), which might be why porphyrin‐based yellow color in feathers is present only in the postnatal period in our species. We hypothesized that this transitional color trait may not only have a role in signaling nestlings' sex (Laczi et al. 2025), but also in signaling quality. In our former study in this study population, we found that nestlings' wing stripe coloration was associated with the rearing environment (Laczi et al. 2026). Specifically, under adverse conditions (i.e., under higher asynchrony with food peak), nestlings developed less yellow wing stripes. The results suggest that variation in porphyrin‐based wings stripe coloration may reflect individual quality. Based on both the present and previous findings, it could be speculated that lower amount of porphyrin pigments may be associated with a worse oxidative state, supporting the signaling value of yellowness. Under these circumstances, the temporary yellowness of the stripe may have at least two functions. First, it is plausible that it can fine‐tune the parental allocation strategy through quality signaling (Romano et al. 2016), and second, it may play roles in other social contexts as well, by signaling sex and immature state (see Laczi et al. 2025 for details). Given the limited knowledge on porphyrin pigments, our conclusions are largely speculative, and further studies are needed to explore the link between porphyrin‐based plumage coloration and oxidative state.

Contrary to our expectations, no association was found between the plumage brightness of any body region and the individuals' oxidative state. This may indicate that the relationship between this color trait and oxidative state is context‐dependent, as demonstrated in Himalayan black bulbuls ( *Hypsipetes leucocephalus nigerrimus* ), where an association was only detectable in one of the two sampling years, specifically during the year in which individuals with higher oxidative stress exhibited brighter plumage (Hung and Li 2015). Alternatively, it is possible that the mechanistic link between reflectance and oxidative state involves processes that have a greater effect on wavelength‐dependent spectral shape (e.g., chroma) rather than overall reflectance intensity.

In conclusion, our results revealed an environmental sensitivity of oxidative state, and associations of varying strength between nestling plumage coloration and oxidative state. The above‐mentioned relationships concerning coloration are consistent with previous findings in nestlings of our population, and the patterns altogether suggest that certain aspects of coloration may convey information about individual quality by reflecting the environmental conditions experienced after hatching (Laczi et al. 2026). In addition, in line with earlier results, our current study indicates that these factors may affect the sexes to different extents. Nevertheless, the exact underlying mechanism and the function of these plumage color traits remain unknown and require further investigations. The specific role of these traits could be revealed by investigating parental allocation strategy in the nest or by conducting studies across different life stages. This could include, for example, analyses extending up to yearling individuals, as certain wing feathers originating from the nestling stage remain until the first breeding period. Finally, further studies should investigate the functional significance of eumelanin to pheomelanin ratios, and especially examine the information content of porphyrin concentrations.

## Author Contributions

**Gabriella Kőmüves:** conceptualization (equal), data curation (equal), formal analysis (equal), investigation (equal), visualization (equal), writing – original draft (equal), writing – review and editing (equal). **Miklós Laczi:** conceptualization (equal), data curation (equal), investigation (equal), writing – review and editing (equal). **Fanni Sarkadi:** data curation (equal), formal analysis (equal), investigation (equal), writing – review and editing (equal). **Gyula Szabó:** investigation (equal), writing – review and editing (equal). **János Török:** investigation (equal), writing – review and editing (equal). **Gergely Hegyi:** conceptualization (equal), formal analysis (equal), funding acquisition (lead), investigation (equal), supervision (lead), writing – review and editing (equal).

## Funding

This research was funded by NKFIH grant K143222 and carried out under a research permit from the regional nature conservation authority (PE‐06/KTF/15049‐5/2022). Miklós Laczi was supported by the János Bolyai Research Scholarship of the Hungarian Academy of Sciences (BO/00213/25/8).

## Conflicts of Interest

The authors declare no conflicts of interest.

## Supporting information


**Table S1:** Results of the analysis of nestlings' wing covert brown chroma. OXY represents plasma antioxidant capacity. Mean ROM was defined as the nest‐level mean amount of reactive oxygen metabolites, whereas ROM deviation indicates the within‐nest deviation from this mean. Both ROM and OXY values were log‐transformed prior to analyses. Effects included in the final models are highlighted in bold.
**Table S2:** Results of the wing stripe saturation (relative height) model after including one influential individual. OXY represents plasma antioxidant capacity. Mean ROM was defined as the nest‐level mean amount of reactive oxygen metabolites, whereas ROM deviation indicates the within‐nest deviation from this mean. Both ROM and OXY values were log‐transformed prior to analyses. Effects included in the final models are highlighted in bold.

## Data Availability

The authors confirm that the data supporting the findings of this study are available in the Figshare data repository at https://doi.org/10.6084/m9.figshare.30550823.v2.

## References

[ece373835-bib-0001] Andersson, M. 1994. Sexual Selection (Vol .72). Princeton University Press.

[ece373835-bib-0002] Arai, E. , M. Hasegawa , T. Makino , et al. 2017. “Physiological Conditions and Genetic Controls of Phaeomelanin Pigmentation in Nestling Barn Swallows.” Behavioral Ecology 28, no. 3: 706–716.

[ece373835-bib-0003] Bezdetnaya, L. , N. Zeghari , I. Belitchenko , et al. 1996. “Spectroscopic and Biological Testing of Photobleaching of Porphyrins in Solutions.” Photochemistry and Photobiology 64, no. 2: 382–386. 8760578 10.1111/j.1751-1097.1996.tb02475.x

[ece373835-bib-0005] Blount, J. D. , N. B. Metcalfe , K. E. Arnold , P. F. Surai , G. L. Devevey , and P. Monaghan . 2003. “Neonatal Nutrition, Adult Antioxidant Defences and Sexual Attractiveness in the Zebra Finch.” Proceedings of the Royal Society of London, Series B: Biological Sciences 270, no. 1525: 1691–1696. 10.1098/rspb.2003.2411 PMC1691426 12964996

[ece373835-bib-0006] Bourgeon, S. , S. Guindre‐Parker , and T. D. Williams . 2011. “Effects of Sibling Competition on Growth, Oxidative Stress, and Humoral Immunity: A Two‐Year Brood‐Size Manipulation.” Physiological and Biochemical Zoology 84, no. 4: 429–437. 21743256 10.1086/661080

[ece373835-bib-0007] Buse, A. , S. J. Dury , R. J. W. Woodburn , C. M. Perrins , and J. E. G. Good . 1999. “Effects of Elevated Temperature on Multi‐Species Interactions: The Case of Pedunculate Oak, Winter Moth and Tits.” Functional Ecology 13: 74–82.

[ece373835-bib-0008] Calhim, S. , P. Adamik , P. Järvistö , et al. 2014. “Heterospecific Female Mimicry in Ficedula Flycatchers.” Journal of Evolutionary Biology 27, no. 3: 660–666. 24494669 10.1111/jeb.12328

[ece373835-bib-0009] Camacho, C. , J. J. Negro , I. Redondo , S. Palacios , and P. Sáez‐Gómez . 2019. “Correlates of Individual Variation in the Porphyrin‐Based Fluorescence of Red‐Necked Nightjars ( *Caprimulgus ruficollis* ).” Scientific Reports 9, no. 1: 19115. 31836769 10.1038/s41598-019-55522-y PMC6910967

[ece373835-bib-0010] Catoni, C. , H. M. Schaefer , and A. Peters . 2008. “Fruit for Health: The Effect of Flavonoids on Humoral Immune Response and Food Selection in a Frugivorous Bird.” Functional Ecology 22: 649–654.

[ece373835-bib-0011] Chaplyhina, A. B. , D. I. Yuzyk , N. O. Savynska , and V. M. Hramma . 2022. “Invertebrates in the Diet of Collared Flycatcher ( *Ficedula albicollis* ) Nestlings in Transformed Forest Ecosystems of North‐Eastern Ukraine: Invertebrates in the Diet of Collared Flycatcher.” Baltic Forestry 28, no. 1: 37–49.

[ece373835-bib-0012] Costantini, D. 2010. “Effects of Diet Quality on Growth Pattern, Serum Oxidative Status, and Corticosterone in Pigeons ( *Columba livia* ).” Canadian Journal of Zoology 88, no. 8: 795–802.

[ece373835-bib-0013] Costantini, D. , S. Casagrande , S. De Filippis , et al. 2006. “Correlates of Oxidative Stress in Wild Kestrel Nestlings ( *Falco tinnunculus* ).” Journal of Comparative Physiology B 176, no. 4: 329–337. 10.1007/s00360-005-0055-6 16344989

[ece373835-bib-0014] Costantini, D. , and G. Dell'Omo . 2006a. “Environmental and Genetic Components of Oxidative Stress in Wild Kestrel Nestlings ( *Falco tinnunculus* ).” Journal of Comparative Physiology B 176, no. 6: 575–579. 10.1007/s00360-006-0080-0 16598486

[ece373835-bib-0015] Costantini, D. , and G. Dell'Omo . 2006b. “Effects of T‐Cell‐Mediated Immune Response on Avian Oxidative Stress.” Comparative Biochemistry and Physiology Part A: Molecular & Integrative Physiology 145, no. 1: 137–142. 10.1016/j.cbpa.2006.06.002 16872854

[ece373835-bib-0016] Costanzo, A. , M. Parolini , G. Bazzi , et al. 2017. “Brood Size, Telomere Length, and Parent‐Offspring Color Signaling in Barn Swallows.” Behavioral Ecology 28, no. 1: 204–211.

[ece373835-bib-0017] D'Alba, L. , C. Van Hemert , K. A. Spencer , et al. 2014. “Melanin‐Based Color of Plumage: Role of Condition and of Feathers' Microstructure.” Integrative and Comparative Biology 54: 633–644. 24987010 10.1093/icb/icu094

[ece373835-bib-0018] De Coster, G. , L. De Neve , S. Verhulst , and L. Lens . 2012. “Maternal Effects Reduce Oxidative Stress in Female Nestlings Under High Parasite Load.” Journal of Avian Biology 43, no. 2: 177–185.

[ece373835-bib-0019] de Zwaan, D. R. , S. Barnes , and K. Martin . 2019. “Plumage Melanism Is Linked to Male Quality, Female Parental Investment and Assortative Mating in an Alpine Songbird.” Animal Behaviour 156: 41–49.

[ece373835-bib-0020] Demongin, L. 2016. Identification Guide to Birds in the Hand. Beauregard‐Vendon.

[ece373835-bib-0021] Ducrest, A. L. , L. Keller , and A. Roulin . 2008. “Pleiotropy in the Melanocortin System, Coloration and Behavioural Syndromes.” Trends in Ecology & Evolution 23, no. 9: 502–510. 18644658 10.1016/j.tree.2008.06.001

[ece373835-bib-0022] Emaresi, G. , P. Bize , and A. Roulin . 2022. “Importance of Melanin‐Based Colouration and Environment in Shaping Intracellular Glutathione Levels in Nestling and Adult Tawny Owls *Strix aluco* .” Journal of Avian Biology 2022, no. 2: e02908.

[ece373835-bib-0023] Ericson, M. B. , S. Grapengiesser , F. Gudmundson , et al. 2003. “A Spectroscopic Study of the Photobleaching of Protoporphyrin IX in Solution.” Lasers in Medical Science 18, no. 1: 56–62. 12627275 10.1007/s10103-002-0254-2

[ece373835-bib-0024] Fargallo, J. A. , T. Laaksonen , E. Korpimäki , and K. Wakamatsu . 2007. “A Melanin‐Based Trait Reflects Environmental Growth Conditions of Nestling Male Eurasian Kestrels.” Evolutionary Ecology 21: 157–171.

[ece373835-bib-0025] Fridolfsson, A. K. , and H. Ellegren . 1999. “A Simple and Universal Method for Molecular Sexing of Non‐Ratite Birds.” Journal of Avian Biology 30: 116–121.

[ece373835-bib-0026] Galván, I. 2017. “Condition‐Dependence of Pheomelanin‐Based Coloration in Nuthatches *Sitta europaea* Suggests a Detoxifying Function: Implications for the Evolution of Juvenile Plumage Patterns.” Scientific Reports 7, no. 1: 9138. 28831177 10.1038/s41598-017-09771-4 PMC5567206

[ece373835-bib-0027] Galván, I. 2022. “Pigment Molecular Composition Reveals Significant Information for Visual Communication.” Functional Ecology 36, no. 11: 2756–2762.

[ece373835-bib-0028] Galván, I. , and C. Alonso‐Alvarez . 2008. “An Intracellular Antioxidant Determines the Expression of a Melanin‐Based Signal in a Bird.” PLoS One 3, no. 10: e3335. 18833330 10.1371/journal.pone.0003335 PMC2556083

[ece373835-bib-0030] Galván, I. , P. R. Camarero , R. Mateo , and J. J. Negro . 2016. “Porphyrins Produce Uniquely Ephemeral Animal Colouration: A Possible Signal of Virginity.” Scientific Reports 6, no. 1: 39210. 27976701 10.1038/srep39210 PMC5156940

[ece373835-bib-0031] Galván, I. , M. D. M. Delgado , P. R. Camarero , R. Mateo , R. Lourenço , and V. Penteriani . 2018. “Feather Content of Porphyrins in Eurasian Eagle Owl ( *Bubo bubo* ) Fledglings Depends on Body Condition and Breeding Site Quality.” Integrative Zoology 13, no. 5: 569–578. 29436755 10.1111/1749-4877.12313

[ece373835-bib-0032] Galván, I. , L. Gangoso , J. M. Grande , et al. 2010. “Antioxidant Machinery Differs Between Melanic and Light Nestlings of Two Polymorphic Raptors.” PLoS One 5, no. 10: e13369. 20976228 10.1371/journal.pone.0013369 PMC2954797

[ece373835-bib-0033] Galván, I. , G. Ghanem , and A. P. Møller . 2012. “Has Removal of Excess Cysteine Led to the Evolution of Pheomelanin? Pheomelanogenesis as an Excretory Mechanism for Cysteine.” BioEssays 34, no. 7: 565–568. 22442057 10.1002/bies.201200017

[ece373835-bib-0034] Galván, I. , Â. Inácio , A. A. Romero‐Haro , and C. Alonso‐Alvarez . 2017. “Adaptive Downregulation of Pheomelanin‐Related Slc7a11 Gene Expression by Environmentally Induced Oxidative Stress.” Molecular Ecology 26, no. 3: 849–858. 27988976 10.1111/mec.13952

[ece373835-bib-0035] Galván, I. , T. A. Mousseau , and A. P. Møller . 2011. “Bird Population Declines due to Radiation Exposure at Chernobyl Are Stronger in Species With Pheomelanin‐Based Coloration.” Oecologia 165: 827–835. 21136083 10.1007/s00442-010-1860-5

[ece373835-bib-0037] Galván, I. , and F. Solano . 2016. “Bird Integumentary Melanins: Biosynthesis, Forms, Function and Evolution.” International Journal of Molecular Sciences 17, no. 4: 520. 27070583 10.3390/ijms17040520 PMC4848976

[ece373835-bib-0038] García‐Campa, J. , W. Müller , and J. Morales . 2023. “Offspring Plumage Coloration as a Condition‐Dependent Signal in the Blue Tit.” Ecology and Evolution 13, no. 2: e9787. 36744078 10.1002/ece3.9787 PMC9889846

[ece373835-bib-0039] Garratt, M. , and R. C. Brooks . 2012. “Oxidative Stress and Condition‐Dependent Sexual Signals: More Than Just Seeing Red.” Proceedings of the Royal Society B: Biological Sciences 279, no. 1741: 3121–3130. 10.1098/rspb.2012.0568 PMC3385731 22648155

[ece373835-bib-0040] Garrido‐Bautista, J. , A. Soria , C. E. Trenzado , et al. 2021. “Oxidative Status of Blue Tit Nestlings Varies With Habitat and Nestling Size.” Comparative Biochemistry and Physiology Part A: Molecular & Integrative Physiology 258: 110986. 10.1016/j.cbpa.2021.110986 34023537

[ece373835-bib-0041] Giordano, M. , D. Costantini , and B. Tschirren . 2015. “Sex‐Specific Effects of Prenatal and Postnatal Nutritional Conditions on the Oxidative Status of Great Tit Nestlings.” Oecologia 177: 123–131. 25376155 10.1007/s00442-014-3100-x

[ece373835-bib-0043] Goldberg, A. , H. Ashenbrucker , G. E. Cartwright , and M. M. Wintrobe . 1956. “Studies on the Biosynthesis of Heme In Vitro by Avian Erythrocytes.” Blood 11, no. 9: 821–833. 13355892

[ece373835-bib-0044] Griffith, S. C. , T. H. Parker , and V. A. Olson . 2006. “Melanin‐Versus Carotenoid‐Based Sexual Signals: Is the Difference Really So Black and Red?” Animal Behaviour 71, no. 4: 749–763.

[ece373835-bib-0045] Guindre‐Parker, S. , and O. P. Love . 2014. “Revisiting the Condition‐Dependence of Melanin‐Based Plumage.” Journal of Avian Biology 45: 29–33.

[ece373835-bib-0046] Hegyi, G. , M. Laczi , G. Szabó , F. Sarkadi , and J. Török . 2023. “Plumage Color Degradation Indicates Reproductive Effort: An Experiment.” Scientific Reports 13, no. 1: 18770. 37907494 10.1038/s41598-023-45348-0 PMC10618437

[ece373835-bib-0047] Hegyi, G. , G. Nagy , and J. Török . 2013. “Reduced Compensatory Growth Capacity in Mistimed Broods of a Migratory Passerine.” Oecologia 172, no. 1: 279–291. 23053241 10.1007/s00442-012-2487-5

[ece373835-bib-0048] Henschen, A. E. , L. A. Whittingham , and P. O. Dunn . 2016. “Oxidative Stress Is Related to Both Melanin‐ and Carotenoid‐Based Ornaments in the Common Yellowthroat.” Functional Ecology 30, no. 5: 749–758.

[ece373835-bib-0049] Hõrak, P. , E. Sild , U. Soomets , T. Sepp , and K. Kilk . 2010. “Oxidative Stress and Information Content of Black and Yellow Plumage Coloration: An Experiment With Greenfinches.” Journal of Experimental Biology 213, no. 13: 2225–2233. 20543121 10.1242/jeb.042085

[ece373835-bib-0050] Hõrak, P. , H. Vellau , I. Ots , and A. P. Møller . 2000. “Growth Conditions Affect Carotenoid‐Based Plumage Coloration of Great Tit Nestlings.” Naturwissenschaften 87, no. 10: 460–464. 11129946 10.1007/s001140050759

[ece373835-bib-0051] Hung, H. Y. , and S. H. Li . 2015. “Brightness of Melanin‐Based Plumage Coloration Is a Cue to Oxidative Stress in Himalayan Black Bulbuls ( *Hypsipetes leucocephalus nigerrimus* ).” Avian Research 6, no. 1: 26.

[ece373835-bib-0052] Ilmonen, P. , D. Hasselquist , Å. Langefors , and J. Wiehn . 2003. “Stress, Immunocompetence and Leukocyte Profiles of Pied Flycatchers in Relation to Brood Size Manipulation.” Oecologia 136, no. 1: 148–154. 12695901 10.1007/s00442-003-1243-2

[ece373835-bib-0053] Isaksson, C. , and S. Andersson . 2008. “Oxidative Stress Does Not Influence Carotenoid Mobilization and Plumage Pigmentation.” Proceedings of the Royal Society B: Biological Sciences 275, no. 1632: 309–314. 10.1098/rspb.2007.1474 PMC2593728 18029305

[ece373835-bib-0054] Jacot, A. , and B. Kempenaers . 2007. “Effects of Nestling Condition on UV Plumage Traits in Blue Tits: An Experimental Approach.” Behavioral Ecology 18, no. 1: 34–40.

[ece373835-bib-0055] Järvinen, A. , and J. Ylimaunu . 1984. “Significance of Egg Size on the Growth of Nestling Pied Flycatchers *Ficedula hypoleuca* .” Annales Zoologici Fennici 21, no. 3: 213–216.

[ece373835-bib-0056] Johnsen, A. , K. Delhey , S. Andersson , and B. Kempenaers . 2003. “Plumage Colour in Nestling Blue Tits: Sexual Dichromatism, Condition Dependence and Genetic Effects.” Proceedings of the Royal Society of London. Series B: Biological Sciences 270, no. 1521: 1263–1270. 10.1098/rspb.2003.2375 PMC1691364 12816639

[ece373835-bib-0057] Kapun, M. , A. Darolová , J. Krištofik , K. Mahr , and H. Hoi . 2011. “Distinct Colour Morphs in Nestling European Bee‐Eaters *Merops apiaster* : Is There an Adaptive Value?” Journal of Ornithology 152: 1001–1005.

[ece373835-bib-0058] Kilner, R. M. 2006. “Function and Evolution of Color in Young Birds.” In Bird Coloration: Function and Evolution, edited by G. E. Hill and K. J. McGraw , 201–232. Harvard University Press.

[ece373835-bib-0059] Kingma, S. A. , I. Szentirmai , T. Székely , et al. 2008. “Sexual Selection and the Function of a Melanin‐Based Plumage Ornament in Polygamous Penduline Tits *Remiz pendulinus* .” Behavioral Ecology and Sociobiology 62: 1277–1288.

[ece373835-bib-0060] Koivula, M. J. , M. Kanerva , J. P. Salminen , M. Nikinmaa , and T. Eeva . 2011. “Metal Pollution Indirectly Increases Oxidative Stress in Great Tit ( *Parus major* ) Nestlings.” Environmental Research 111, no. 3: 362–370. 21295293 10.1016/j.envres.2011.01.005

[ece373835-bib-0061] Kose, M. , and A. P. Møller . 1999. “Sexual Selection, Feather Breakage and Parasites: The Importance of White Spots in the Tail of the Barn Swallow ( *Hirundo rustica* ).” Behavioral Ecology and Sociobiology 45, no. 6: 430–436.

[ece373835-bib-0062] Krebs, E. A. , and D. A. Putland . 2004. “Chic Chicks: The Evolution of Chick Ornamentation in Rails.” Behavioral Ecology 15, no. 6: 946–951.

[ece373835-bib-0063] Kuznetsova, A. , P. B. Brockhoff , and R. H. B. Christensen . 2017. “lmerTest Package: Tests in Linear Mixed Effects Models.” Journal of Statistical Software 82, no. 13: 1–26.

[ece373835-bib-0064] Laczi, M. , G. Hegyi , D. Kötél , T. Csizmadia , P. Lőw , and J. Török . 2019. “Reflectance in Relation to Macro‐ and Nanostructure in the Crown Feathers of the Great Tit ( *Parus major* ).” Biological Journal of the Linnean Society 127, no. 1: 113–124.

[ece373835-bib-0065] Laczi, M. , G. Herczeg , F. Sarkadi , et al. 2025. “Nestling Plumage Colour Variation in a Sexually Dichromatic Hole‐Nesting Passerine Bird—Potential Functions and Mechanisms.” Ecology and Evolution 15, no. 4: e71152. 40177693 10.1002/ece3.71152 PMC11962215

[ece373835-bib-0066] Laczi, M. , G. Herczeg , F. Sarkadi , et al. 2026. “Information Content of Persistent Plumage Colour Traits in *Ficedula albicollis* Nestlings.” Ornithology ukaf070: 1–11.

[ece373835-bib-0067] Larcombe, S. D. , W. Mullen , L. Alexander , and K. E. Arnold . 2010. “Dietary Antioxidants, Lipid Peroxidation and Plumage Colouration in Nestling Blue Tits *Cyanistes caeruleus* .” Naturwissenschaften 97: 903–913. 20838757 10.1007/s00114-010-0708-5

[ece373835-bib-0068] Lee, S. , K. H. Han , Y. Nakamura , et al. 2013. “Dietary L‐Cysteine Improves the Antioxidative Potential and Lipid Metabolism in Rats Fed a Normal Diet.” Bioscience, Biotechnology, and Biochemistry 77, no. 7: 1430–1434. 23832363 10.1271/bbb.130083

[ece373835-bib-0069] Lessells, C. M. 2002. “Parentally Biased Favouritism: Why Should Parents Specialize in Caring for Different Offspring?” Philosophical Transactions of the Royal Society of London. Series B: Biological Sciences 357, no. 1419: 381–403. 11958706 10.1098/rstb.2001.0928 PMC1692952

[ece373835-bib-0070] López‐Arrabé, J. , A. Cantarero , L. Pérez‐Rodríguez , A. Palma , and J. Moreno . 2016. “Oxidative Stress in Early Life: Associations With Sex, Rearing Conditions, and Parental Physiological Traits in Nestling Pied Flycatchers.” Physiological and Biochemical Zoology 89, no. 2: 83–92. 27082719 10.1086/685476

[ece373835-bib-0071] Losdat, S. , F. Helfenstein , J. D. Blount , and H. Richner . 2014. “Resistance to Oxidative Stress Shows Low Heritability and High Common Environmental Variance in a Wild Bird.” Journal of Evolutionary Biology 27, no. 9: 1990–2000. 25040169 10.1111/jeb.12454

[ece373835-bib-0072] Losdat, S. , F. Helfenstein , B. Gaude , and H. Richner . 2010. “Effect of Sibling Competition and Male Carotenoid Supply on Offspring Condition and Oxidative Stress.” Behavioral Ecology 21, no. 6: 1271–1277.

[ece373835-bib-0073] Lyon, B. E. , J. M. Eadie , and L. D. Hamilton . 1994. “Parental Choice Selects for Ornamental Plumage in American Coot Chicks.” Nature 371: 240–243.

[ece373835-bib-0074] Markó, G. , D. Costantini , G. Michl , and J. Török . 2011. “Oxidative Damage and Plasma Antioxidant Capacity in Relation to Body Size, Age, Male Sexual Traits and Female Reproductive Performance in the Collared Flycatcher ( *Ficedula albicollis* ).” Journal of Comparative Physiology B 181: 73–81. 10.1007/s00360-010-0502-x 20677008

[ece373835-bib-0075] Marri, V. , and H. Richner . 2014. “Differential Effects of Vitamins E and C and Carotenoids on Growth, Resistance to Oxidative Stress, Fledging Success and Plumage Colouration in Wild Great Tits.” Journal of Experimental Biology 217, no. 9: 1478–1484. 24436384 10.1242/jeb.096826

[ece373835-bib-0076] Marton, A. , C. I. Vágási , O. Vincze , et al. 2022. “Oxidative Physiology Is Weakly Associated With Pigmentation in Birds.” Ecology and Evolution 12, no. 8: e9177. 35979521 10.1002/ece3.9177 PMC9366753

[ece373835-bib-0077] McGlothlin, J. W. , D. L. Duffy , J. L. Henry‐Freeman , and E. D. Ketterson . 2007. “Diet Quality Affects an Attractive White Plumage Pattern in Dark‐Eyed Juncos ( *Junco hyemalis* ).” Behavioral Ecology and Sociobiology 61: 1391–1399.

[ece373835-bib-0078] McGraw, K. J. 2007. “Dietary Mineral Content Influences the Expression of Melanin‐Based Ornamental Coloration.” Behavioral Ecology 18, no. 1: 137–142.

[ece373835-bib-0079] McGraw, K. J. 2008. “An Update on the Honesty of Melanin‐Based Color Signals in Birds.” Pigment Cell & Melanoma Research 21, no. 2: 133–138. 18426406 10.1111/j.1755-148X.2008.00454.x

[ece373835-bib-0081] McGraw, K. J. , E. A. Mackillop , J. Dale , and M. E. Hauber . 2002. “Different Colors Reveal Different Information: How Nutritional Stress Affects the Expression of Melanin‐ and Structurally Based Ornamental Plumage.” Journal of Experimental Biology 205, no. 23: 3747–3755. 12409501 10.1242/jeb.205.23.3747

[ece373835-bib-0082] McGraw, K. J. , R. J. Safran , and K. Wakamatsu . 2005. “How Feather Colour Reflects Its Melanin Content.” Functional Ecology 19, no. 5: 816–821.

[ece373835-bib-0083] McKinnon, E. A. , and R. J. Robertson . 2008. “The Signal Function of a Melanin‐Based Plumage Ornament in Golden‐Winged Warblers.” Wilson Journal of Ornithology 120, no. 2: 366–370.

[ece373835-bib-0121] McNeil, K. A. , I. Newman , and F. J. Kelly . 1996. Testing Research Hypotheses With the General Linear Model. SIU Press.

[ece373835-bib-0085] Mennill, D. J. , S. M. Doucet , R. Montgomerie , and L. M. Ratcliffe . 2003. “Achromatic Color Variation in Black‐Capped Chickadees, *Poecile atricapilla*: Black and White Signals of Sex and Rank.” Behavioral Ecology and Sociobiology 53, no. 6: 350–357.

[ece373835-bib-0086] Metcalfe, N. B. , and C. Alonso‐Alvarez . 2010. “Oxidative Stress as a Life‐History Constraint: The Role of Reactive Oxygen Species in Shaping Phenotypes From Conception to Death.” Functional Ecology 24, no. 5: 984–996.

[ece373835-bib-0088] Moan, J. 1988. “A Change in the Quantum Yield of Photoinactivation of Cells Observed During Photodynamic Treatment.” Lasers in Medical Science 3, no. 1: 93–97.

[ece373835-bib-0089] Musgrove, A. B. , and K. L. Wiebe . 2016. “Condition‐Dependent Expression of Carotenoid‐ and Melanin‐Based Plumage Colour of Northern Flicker Nestlings Revealed by Manipulation of Brood Size.” Journal of Avian Biology 47, no. 2: 176–184.

[ece373835-bib-0090] Nicholls, J. A. , M. C. Double , D. M. Rowell , and R. D. Magrath . 2000. “The Evolution of Cooperative and Pair Breeding in Thornbills Acanthiza (Pardalotidae).” Journal of Avian Biology 31, no. 2: 165–176.

[ece373835-bib-0091] Örnborg, J. , S. Andersson , S. C. Griffith , and B. C. Sheldon . 2002. “Seasonal Changes in a Ultraviolet Structural Colour Signal in Blue Tits, *Parus caeruleus* .” Biological Journal of the Linnean Society 76, no. 2: 237–245.

[ece373835-bib-0092] Pardal, S. , J. A. Alves , P. G. Mota , and J. A. Ramos . 2018. “Dressed to Impress: Breeding Plumage as a Reliable Signal of Innate Immunity.” Journal of Avian Biology 49, no. 7: e01579.

[ece373835-bib-0093] R Core Team . 2023. R: A Language and Environment for Statistical Computing. R Foundation for Statistical Computing. https://www.R‐project.org/.

[ece373835-bib-0094] Riesz, J. J. 2007. “The Spectroscopic Properties of Melanin.” PhD thesis, University of Queensland, Queensland, Australia.

[ece373835-bib-0095] Rodríguez‐Martínez, S. , R. Márquez , Â. Inácio , and I. Galván . 2019. “Changes in Melanocyte RNA and DNA Methylation Favour Pheomelanin Synthesis and May Avoid Systemic Oxidative Stress After Dietary Cysteine Supplementation in Birds.” Molecular Ecology 28, no. 5: 1030–1042. 30661260 10.1111/mec.15024

[ece373835-bib-0096] Romano, A. , G. Bazzi , M. Caprioli , et al. 2016. “Nestling Sex and Plumage Color Predict Food Allocation by Barn Swallow Parents.” Behavioral Ecology 27, no. 4: 1198–1205.

[ece373835-bib-0098] Rosivall, B. , J. Török , D. Hasselquist , and S. Bensch . 2004. “Brood Sex Ratio Adjustment in Collared Flycatchers ( *Ficedula albicollis* ): Results Differ Between Populations.” Behavioral Ecology and Sociobiology 56: 346–351.

[ece373835-bib-0099] Roulin, A. , B. Almasi , K. S. Meichtry‐Stier , and L. Jenni . 2011. “Eumelanin‐ and Pheomelanin‐Based Colour Advertise Resistance to Oxidative Stress in Opposite Ways.” Journal of Evolutionary Biology 24, no. 10: 2241–2247. 21745253 10.1111/j.1420-9101.2011.02353.x

[ece373835-bib-0100] Roulin, A. , C. Dijkstra , C. Riols , and A. L. Ducrest . 2001. “Female‐ and Male‐Specific Signals of Quality in the Barn Owl.” Journal of Evolutionary Biology 14, no. 2: 255–266.

[ece373835-bib-0101] San‐Jose, L. M. , and A. Roulin . 2018. “Toward Understanding the Repeated Occurrence of Associations Between Melanin‐Based Coloration and Multiple Phenotypes.” American Naturalist 192, no. 2: 111–130. 10.1086/698010 30016163

[ece373835-bib-0102] Shawkey, M. D. , and L. D'Alba . 2017. “Interactions Between Colour‐Producing Mechanisms and Their Effects on the Integumentary Colour Palette.” Philosophical Transactions of the Royal Society, B: Biological Sciences 372, no. 1724: 20160536. 10.1098/rstb.2016.0536 PMC5444072 28533449

[ece373835-bib-0103] Shawkey, M. D. , and G. E. Hill . 2005. “Carotenoids Need Structural Colours to Shine.” Biology Letters 1, no. 2: 121–124. 17148144 10.1098/rsbl.2004.0289 PMC1626226

[ece373835-bib-0104] Siefferman, L. , and G. E. Hill . 2007. “The Effect of Rearing Environment on Blue Structural Coloration of Eastern Bluebirds ( *Sialia sialis* ).” Behavioral Ecology and Sociobiology 61: 1839–1846. 19655039 10.1007/s00265-007-0416-0 PMC2719904

[ece373835-bib-0105] Siefferman, L. , M. D. Shawkey , R. Bowman , and G. E. Woolfenden . 2008. “Juvenile Coloration of Florida Scrub‐Jays ( *Aphelocoma coerulescens* ) is Sexually Dichromatic and Correlated With Condition.” Journal of Ornithology 149: 357–363.

[ece373835-bib-0106] Siitari, H. , and E. Huhta . 2002. “Individual Color Variation and Male Quality in Pied Flycatchers ( *Ficedula hypoleuca* ): A Role of Ultraviolet Reflectance.” Behavioral Ecology 13, no. 6: 737–741.

[ece373835-bib-0107] Stoffel, M. A. , S. Nakagawa , and H. Schielzeth . 2017. “rptR: Repeatability Estimation and Variance Decomposition by Generalized Linear Mixed‐Effects Models.” Methods in Ecology and Evolution 8, no. 11: 1639–1644.

[ece373835-bib-0108] Surmacki, A. 2008. “Preen Waxes Do Not Protect Carotenoid Plumage From Bleaching by Sunlight.” Ibis 150, no. 2: 335–341.

[ece373835-bib-0109] Surmacki, A. , M. Liu , A. Mercadante , and G. E. Hill . 2011. “Effect of Feather Abrasion on Structural Coloration in Male Eastern Bluebirds *Sialia sialis* .” Journal of Avian Biology 42, no. 6: 514–521.

[ece373835-bib-0110] Surmacki, A. , and J. K. Nowakowski . 2007. “Soil and Preen Waxes Influence the Expression of Carotenoid‐Based Plumage Coloration.” Naturwissenschaften 94, no. 10: 829–835. 17541535 10.1007/s00114-007-0263-x

[ece373835-bib-0111] Surmacki, A. , J. Stępniewski , and M. Stępniewska . 2015. “Juvenile Sexual Dimorphism, Dichromatism and Condition‐Dependent Signaling in a Bird Species With Early Pair Bonds.” Journal of Ornithology 156: 65–73.

[ece373835-bib-0112] Számadó, S. 2011. “The Cost of Honesty and the Fallacy of the Handicap Principle.” Animal Behaviour 81, no. 1: 3–10.

[ece373835-bib-0113] Szigeti, B. , J. Török , G. Hegyi , et al. 2007. “Egg Quality and Parental Ornamentation in the Blue Tit *Parus caeruleus* .” Journal of Avian Biology 38, no. 1: 105–112.

[ece373835-bib-0114] Tran, M. L. , B. J. Powell , and P. Meredith . 2006. “Chemical and Structural Disorder in Eumelanins: A Possible Explanation for Broadband Absorbance.” Biophysical Journal 90, no. 3: 743–752. 16284264 10.1529/biophysj.105.069096 PMC1367100

[ece373835-bib-0115] Van de Pol, M. , and J. Wright . 2009. “A Simple Method for Distinguishing Within‐Versus Between‐Subject Effects Using Mixed Models.” Animal Behaviour 77, no. 3: 753–758.

[ece373835-bib-0116] Wan, X. L. , G. Y. Ju , L. Xu , H. M. Yang , and Z. Y. Wang . 2019. “Dietary Selenomethionine Increases Antioxidant Capacity of Geese by Improving Glutathione and Thioredoxin Systems.” Poultry Science 98, no. 9: 3763–3769. 10.3382/ps/pez066 30815679

[ece373835-bib-0117] Wickham, H. 2016. ggplot2: Elegant Graphics for Data Analysis. Springer‐Verlag.

[ece373835-bib-0118] Woods, J. S. , and C. A. Calas . 1989. “Iron Stimulation of Free Radical‐Mediated Porphyrinogen Oxidation by Hepatic and Renal Mitochondria.” Biochemical and Biophysical Research Communications 160, no. 1: 101–108. 2540739 10.1016/0006-291x(89)91626-4

[ece373835-bib-0119] Wu, G. , J. R. Lupton , N. D. Turner , Y. Z. Fang , and S. Yang . 2004. “Glutathione Metabolism and Its Implications for Health.” Journal of Nutrition 134, no. 3: 489–492. 14988435 10.1093/jn/134.3.489

[ece373835-bib-0120] Zahavi, A. 1977. “The Cost of Honesty (Further Remarks on the Handicap Principle).” Journal of Theoretical Biology 67, no. 3: 603–605. 904334 10.1016/0022-5193(77)90061-3

