# Supplementary material for: Variation in the Oxidative State of Collared Flycatcher (Ficedula albicollis) Nestlings and Its Association With Their Plumage Coloration
Source: Ecol Evol. 2026 Jun 9;16(6):e73835. doi: 10.1002/ece3.73835 (PMC13249541; doi:10.1002/ece3.73835)
Supplement: Supplementary file 1 — Table S1: Results of the analysis of nestlings' wing covert brown chroma. OXY represents plasma antioxidant capacity. Mean ROM was defined as the nest‐level mean amount of reactive oxygen metabolites, whereas ROM deviation indicates the within‐nest deviation from this mean. Both ROM and OXY values were log‐transformed prior to analyses. Effects included in the final models are highlighted in bold. Table S2: Results of the wing stripe saturation (relative height) model after including one influential individual. OXY represents plasma antioxidant capacity. Mean ROM was defined as the nest‐level mean amount of reactive oxygen metabolites, whereas ROM deviation indicates the within‐nest deviation from this mean. Both ROM and OXY values were log‐transformed prior to analyses. Effects included in the final models are highlighted in bold. [file ECE3-16-e73835-s001.docx]

Supplementary material

**Table 1:** Results of the analysis of nestlings’ wing covert brown chroma. OXY represents plasma antioxidant capacity. Mean ROM was defined as the nest-level mean amount of reactive oxygen metabolites, whereas ROM deviation indicates the within-nest deviation from this mean. Both ROM and OXY values were log-transformed prior to analyses. Effects included in the final models are highlighted in bold.

|  | **ß** ± **SE** | **df1** | **df2** | **F** |
| --- | --- | --- | --- | --- |
| **sex** | **0.011 ± 0.002** | **1** | **196.98** | **20.26 ***** |
| **hatching date** | 0.000 ± 0.001 | 1 | 45.09 | 0.59 |
| **OXY** | -0.003 ± 0.008 | 1 | 207.99 | 0.13 |
| **mean ROM** | 0.011 ± 0.019 | 1 | 44.43 | 0.33 |
| **ROM deviation** | **-0.364 ± 0.141** | **1** | **202.49** | **6.67 *** |
| **sex * OXY** | 0.014 ± 0.017 | 1 | 198.71 | 0.68 |
| **sex * mean ROM** | 0.002 ± 0.032 | 1 | 190.22 | 0.00 |
| **sex * ROM deviation** | 0.015 ± 0.029 | 1 | 211.00 | 0.26 |
| **OXY * mean ROM** | 0.090 ± 0.112 | 1 | 202.22 | 0.65 |
| **OXY * ROM deviation** | **0.196 ± 0.077** | **1** | **202.74** | **6.54 *** |
| **sex * OXY * mean ROM** | 0.085 ± 0.112 | 2 | 199.73 | 0.52 |
| **sex * OXY * ROM deviation** | 0.006 ± 0.015 | 1 | 210.99 | 0.16 |

† P<0.10, * P<0.05, ** P<0.01, *** P<0.001

**Table 2:** Results of the wing stripe saturation (relative height) model after excluding one influential individual. OXY represents plasma antioxidant capacity. Mean ROM was defined as the nest-level mean amount of reactive oxygen metabolites, whereas ROM deviation indicates the within-nest deviation from this mean. Both ROM and OXY values were log-transformed prior to analyses. Effects included in the final models are highlighted in bold.

|  | **ß** ± **SE** | **df1** | **df2** | **F** |
| --- | --- | --- | --- | --- |
| **sex** | **0.045 ± 0.020** | **1** | **188.98** | **4.85 *** |
| **hatching date** | **0.014 ± 0.006** | **1** | **43.37** | **5.54 *** |
| **OXY** | -0.128 ± 0.070 | 1 | 197.24 | 3.36 † |
| **mean ROM** | -0.200 ± 0.223 | 1 | 43.32 | 0.81 |
| **ROM deviation** | -0.061 ± 0.108 | 1 | 171.92 | 0.32 |
| **sex * OXY** | -0.112 ± 0.139 | 1 | 187.13 | 0.65 |
| **sex * mean ROM** | 0.145 ± 0.266 | 1 | 185.14 | 0.30 |
| **sex * ROM deviation** | -0.376 ± 0.238 | 1 | 207.54 | 2.49 |
| **OXY * mean ROM** | -0.932 ± 0.929 | 1 | 192.30 | 1.01 |
| **OXY * ROM deviation** | -0.053 ± 0.646 | 1 | 188.63 | 0.01 |
| **sex * OXY * mean ROM** | -0.910 ± 0.934 | 2 | 189.29 | 0.55 |
| **sex * OXY * ROM deviation** | 0.069 ± 0.648 | 2 | 196.77 | 1.71 |

† P<0.10, * P<0.05, ** P<0.01, *** P<0.001
